# Retraction: MicroRNA-19a and microRNA-19b promote the malignancy of clear cell renal cell carcinoma through targeting the tumor suppressor RhoB

**DOI:** 10.1371/journal.pone.0341484

**Published:** 2026-01-26

**Authors:** The PLOS One Editors

Following the publication of this article [1] concerns were raised about results presented in Figs 1, 3, 4, and 5. Specifically,

- The following blot results appear similar despite representing different experimental conditions:
  - Lanes 1-7 of the Fig 1E RhoB and β-actin panels in [1] and Fig 1B RhoB and β-actin panels in [2, retracted in 3],
  - Fig 5B Cleaved caspase-9 left panel and Fig S2 RhoB lanes 3-4.
  - Fig 5B β-actin left panel and Fig S2 β-actin lanes 2-3.
- The following panels appear to partially overlap:
  - Fig 3D miR-19b mimics and Fig 3F RhoB siRNA.
  - Fig 4B mir-19a inhibitor and Fig 4B mir-19b inhibitor.
- The Fig 4A mir-19a inhibitor 24h panel appears similar to the Fig 4A inhibitor control 12h panel.

First author SN replied to the above concerns stating that image preparation errors were made during the preparation of Figs 1 and S2, and that experimental images used for Figs 3 and 4 were inadvertently mixed leading to incorrect use of images. They provided individual level data including underlying QRT-PCR results, but the original triplicate image data underlying the published figures was not provided for editorial review. First author SN offered to repeat the experiments, but PLOS does not consider that repeat experiments alone are sufficient to resolve the concerns with the figures as published.

In light of the above concerns, which call into question the reliability of the published results, the *PLOS One* Editors retract this article.

SN responded but expressed neither agreement nor disagreement with the editorial decision. XM, YZ, YNL, XC, HG, YY, KL, and XZ either did not respond directly or could not be reached.
